# Self-control study of multi-omics in identification of microenvironment characteristics in urine of uric acid stone

**DOI:** 10.1038/s41598-024-76054-0

**Published:** 2024-10-24

**Authors:** Shang Xu, Zhi-Long Liu, Tian-Wei Zhang, Bin Li, Xin-Ning Wang, Wei Jiao

**Affiliations:** Department of Urology, The Affiliated Hospital of Qingdao University, Qingdao, 266000 Shandong Province China

**Keywords:** Urolithiasis, Proteomics, Metabolomics, Urine, Liquid chromatography-tandem mass spectrometry, Metabolomics, Proteomics, Renal calculi

## Abstract

The aim of this study is to perform proteomic and metabolomic analyses in bilateral renal pelvis urine of patients with unilateral uric acid kidney stones to identify the specific urinary environment associated with uric acid stone formation. Using cystoscopy-guided insertion of ureteral catheters, bilateral renal pelvis urine samples are collected. Liquid chromatography-tandem mass spectrometry (LC-MS/MS) is employed to identify differentially expressed proteins and metabolites in the urine environment. Differentially expressed proteins and metabolites are further analyzed for their biological functions and potential metabolic pathways through Gene Ontology (GO) analysis and Kyoto Encyclopedia of Genes and Genomes (KEGG) enrichment analysis. In the urine from the stone-affected side, eight differential proteins were significantly upregulated, and six metabolites were dysregulated. The uric acid stone urinary environment showed an excess of α-ketoisovaleric acid and 3-methyl-2-oxovaleric acid, which may contribute to the acidification of the urine. Functional and pathway analyses indicate that the dysregulated metabolites are mainly associated with insulin resistance and branched chain amino acid metabolism.

**Supplementary Information:**

The online version contains supplementary material available at 10.1038/s41598-024-76054-0.

## Introduction

Kidney stones are a chronic kidney disease with high incidence and recurrence rates, causing great pain and medical burden to patients^1,2^. Uric acid stones are a relatively special type of urinary stones because they do not contain calcium and have unique physical properties. They are one of the few types of stones that can dissolve in the body and have been widely studied throughout history^3^. Uric acid is a product of purine metabolism; it is primarily derived from endogenous sources, with dietary purines generally contributing little to uric acid production. Uric acid cannot be broken down in the human body and is mainly excreted through the kidneys^4,5^. Due to various factors, the uric acid concentration rises in the renal pelvis and then crystallizes out, further aggregating to form uric acid stones.

High-throughput biotechnologies have facilitated the collection of omics data, shedding light on the pathogenesis, biomarkers, and potential therapeutic targets for many diseases. The advancement of metabolomics and proteomics has opened new avenues for investigating the pathogenic mechanisms underlying kidney stones^6^. Previous research has conducted multi-omic analyses of blood and urine components in individuals with kidney stones. However, factors such as diet, lifestyle, systemic disease, and even the time of collection of urine have a greater impact on the metabolites or urinary proteins in an individual’s urine. To minimize these errors, traditional metabolomics and proteomics require large sample sizes and strict control over variables such as the subjects’ diet^7,8^. In our clinical practice, we have observed that some patients have a high recurrence rate of unilateral renal uric acid stones, and some patients even form unilateral staghorn calculi in one kidney while the other kidney remains stone-free. A large cross-sectional study involving 10,281 participants in rural China showed that among all the participants, 4.9% (507) had unilateral renal stones, and 0.7% (75) had bilateral renal stones^9^. This observation has drawn our attention. To explore the unique urinary environment involved in the formation and development of uric acid stones, we conducted proteomic and metabolomic analyses on the bilateral renal pelvis urine collected from unilateral uric acid stone patients.

## Method

### Patients

From May 2023 to July 2023, a total of nine patients (4 males and 5 females) diagnosed with unilateral kidney stones via Computed Tomography (CT) scan were recruited at the Affiliated Hospital of Qingdao University. Enrollment criteria included: (1) Age range of 18 to 80 years; (2) Presence of unilateral kidney stones, with a maximum stone diameter exceeding 1 cm on CT scans; (3) Postoperative stone analysis by infrared spectroscopy confirming that uric acid stone was the only component. Exclusion criteria were: (1) Patients with concurrent malignant tumors, hematologic disorders, metabolic diseases, or rheumatic immune diseases; (2) Individuals with urinary diversion methods in place, such as those who have undergone total cystectomy or kidney transplantation; (3) Patients with other renal pathologies, including renal tumors, previous kidney surgeries, solitary kidneys, renal malformations (e.g., horseshoe kidney, duplicated kidney), or acute and chronic renal diseases, including end-stage renal failure; (4) Those with severe urinary tract infections or a fever exceeding 37.5 °C; (5) Patients experiencing urinary tract obstructions and severe hydronephrosis.

All samples were collected prior to any medical or surgical intervention. Informed consent was secured from each participant before sample collection. The study was conducted following ethical approval by the Bioethics Committee of the Affiliated Hospital of Qingdao University.

### Sample collection and preparation

Before the lithotripsy operation, 10 ml of urine was obtained from each renal pelvis after bilateral ureteral catheterization with F5 ureteral catheter through cystoscope. The urine was centrifuged at 1000 rpm for 15 min and then stored at − 80 °C. Then the lithotripsy continues. 300 µL of 8 M urea was added to the sample and the protease inhibitor was added at 10% of the lysate volume. After centrifuging at 14,100×*g* for 20 min, the supernatant was collected. The protein concentration was determined using Bradford method, rest was frozen to − 80 °C.

### Mass spectrometry conditions for urinary protein profile

For spectral library generation, samples were fractionated using a high pH reversed-phase fractionator. Mass spectrometry were subjected to liquid chromatography-tandem mass spectrometry (LC-MS/MS) using a 150 μm I.D. 25 cm C18 column, 1.9 μm particle size and a 80 min gradient: 8–95% solvent B (solvent A: 99.9% water, 0.1% formic acid(FA); solvent B: 80% acetonitrile, 0.1% FA). The mass spectrometer was operated on a quadrupole Orbitrap mass spectrometer (Q Exactive HF-X, Thermo Fisher Scientific, MA, USA) coupled to an EASY nLC 1200 ultra-high pressure system (Thermo Fisher Scientific) via a nano-electrospray ion source.

### Data analysis of differential expression proteins (DEPs)

Gene Ontology (GO) was conducted using the interproscan-5 program against the non-redundant protein database, and the KEGG (Kyoto Encyclopedia of Genes and Genomes)^10–12^ databases was used to analyze the protein family and pathway. Upregulated proteins were set as having a fold change (FC) ≥ 1.5, and downregulated proteins were set as having a FC ≤ 0.67. The protein quantitative values in the comparisons were statistically analyzed using the paired t-test or Wilcoxon test, with a P-value of ≤ 0.05 considered as statistically significant. When P value < 0.05 and FC > 1.5 or FC < 0.67, we consider the protein to have significant differential expression.

### Mass spectrometry conditions for urinary metabolic profile

Mass spectrometric detection of metabolites was performed on Orbitrap Exploris 120 (Thermo Fisher Scientific, USA) with ESI ion source. Simultaneous MS1 and MS/MS (Full MS-ddMS2 mode, data-dependent MS/MS) acquisition was used. The parameters were as follows: sheath gas pressure, 40 arb; aux gas flow, 10 arb; spray voltage, 3.50 kV and − 2.50 kV for ESI(+) and ESI(−), respectively; capillary temperature, 325 °C; MS1 range, m/z 100–1000; MS1 resolving power, 60,000 full width at half maxima (FWHM); number of data dependant scans per cycle, 4; MS/MS resolving power, 15,000 FWHM; normalized collision energy, 30%; dynamic exclusion time, automatic.

### Data analysis of metabolite and differential metabolites (DMs)

Upregulated metabolites were set as having a FC ≥ 1.2, and downregulated proteins were set as having a FC ≤ 0.83. The metabolites quantitative values in the comparisons were statistically analyzed using the paired t-test or Wilcoxon test, with a P-value of ≤ 0.05 considered as statistically significant. Finally, biomarker metabolites were screened by combining P value and FC. By default, when P value < 0.05 and FC > 1.2 or FC < 0.83, we consider that metabolite are considered to have significant differential expression.

## Result

### Demographic and clinical data of the studied subjects

This study included a total of 9 patients diagnosed with unilateral kidney stones. Bilateral renal pelvis urine was collected from these individuals. Detailed participant demographics and clinical characteristics are presented in Table 1. All analyzed stones primarily consisted of uric acid anhydrous or uric acid dihydrate.

**Table 1:** Demographic and clinical data of the studied subjects.

Number	Age (y)	Gender	BMI (kg/m^2^)	Diabetes mellitus	Hypertension	Plasma Cr (µmol/L)	Plasma uric acid (µmol/L)	Number of stone	Stone size (cm^2^)	Hydronephrosis	Stone side	Urine PH
1	56	Female	23.8	No	Yes	52	458	Single	3.5 × 2.4	No	Right	5.5
2	74	Female	22.2	Yes	Yes	72	301	Multiple	3.0 × 2.5	Mild	Right	5.5
3	75	Male	22.7	No	No	79	435	Staghorn	4.0 × 3.0	No	Right	5.5
4	54	Male	31.8	No	Yes	64	550	Multiple	2.5 × 0.6	No	Left	5.0
5	30	Female	35.2	No	No	77	643	Single	3.0 × 1.0	No	Left	5.0
6	63	Male	27	No	Yes	65	546	Single	4.5 × 3.0	No	Right	5.0
7	37	Male	27.2	No	No	61	494	Single	3.5 × 2.5	No	Left	5.0
8	62	Female	29.7	No	No	48	361	Single	2.0 × 2.0	No	Left	6.5
9	51	Female	19.2	Yes	No	45	190	Single	4.0 × 2.0	No	Left	5.5

### Proteomic analysis of urine samples

**Fig. 1:**
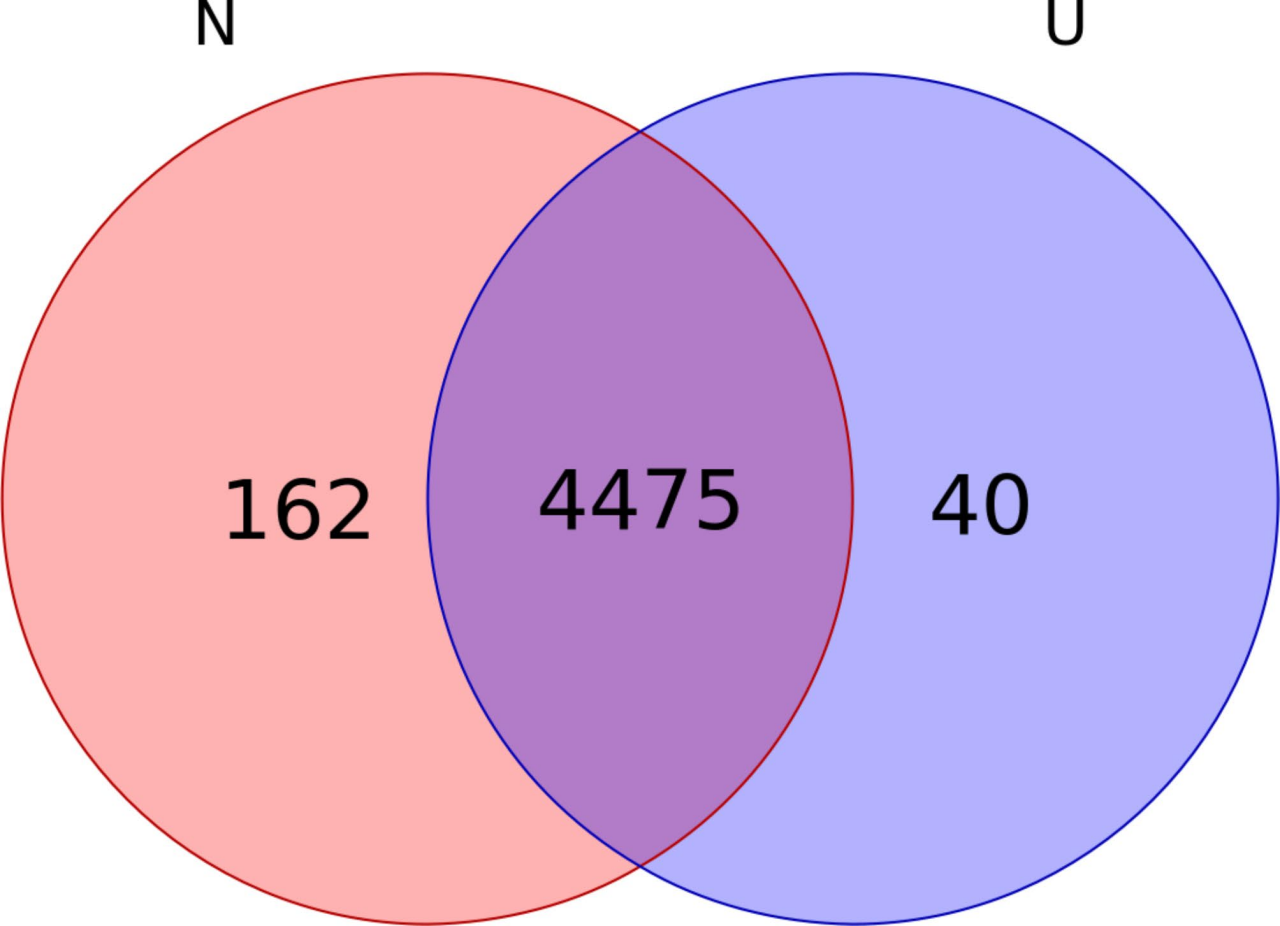
Number of proteins found in Non-stone group, Uric acid stone group.

A total of 4677 proteins were identified by LC-MS/MS analysis of bilateral renal pelvis urine from the nine patients. In this cohort, 4637 proteins were detected in the non-stone group and 4515 in the uric acid stone group, as shown in Fig. 1. Utilizing criteria of fold change (FC) > 1.5 or < 0.67 and a P-value < 0.05, we distinguished 8 up-regulated differential proteins between the groups, as detailed in Table 2. The relative quantification of the 8 differential proteins in the 9 paired samples is shown in Figure S1.

**Table 2:** List of differential proteins in the urine of bilateral renal pelvis in patients with unilateral kidney stones.

Protein name	Abbreviation	Accession	Mean FC	*P*-value	Expression
Eosinophil peroxidase	EPX	P11678	110.7552	0.008	Up
Beta/gamma crystallin domain-containing protein 2	CRYBG2	Q8N1P7	8.309659	0.008	Up
Cholinesterase	BCHE	P06276	8.24964	0.005	Up
Cell division cycle 5-like protein	CDC5L	Q99459	7.09554	0.009	Up
Prohibitin 1	PHB1	P35232	6.794627	0.001	Up
Ficolin-3	FCN3	O75636	5.098481	0.018	Up
Disintegrin and metalloproteinase domain-containing protein 10	ADAM10	O14672	4.222316	0.036	Up
26 S proteasome non-ATPase regulatory subunit 7	PSMD7	P51665	3.691812	0.010	Up

*FC* Fold change.

### Gene ontology (GO) analysis of DEPs

**Fig. 2:**
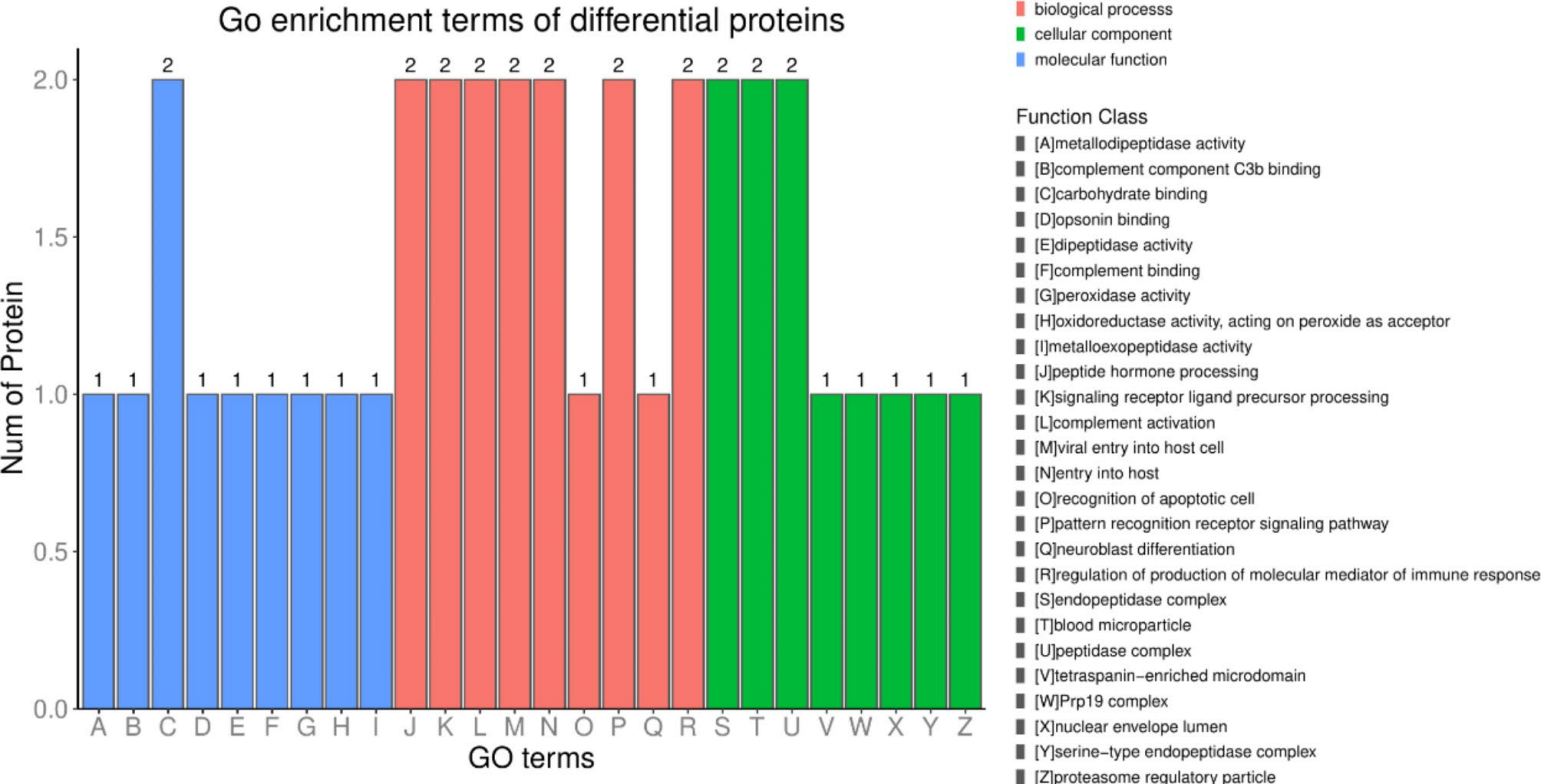
GO analysis of the 8 DEPs for functional classification. Red, green and blue bars represent biological processes, cellular components, and molecular functions, respectively. The vertical axis represents the number of DEPs.

To explore the possible biological functions of dysregulation proteins, we used the InterProScan-5 program against the non-redundant protein database. Results demonstrated that the most enriched GO terms were related to cellular component (endopeptidase complex, blood microparticle, peptidase complex), biological process (peptide hormone processing, signaling receptor ligand precursor processing, complement activation, viral entry into host cell, entry into host, pattern recognition receptor signaling pathway, regulation of the production of molecular mediators of immune response), and molecular function (carbohydrate binding) (Fig. 2).

### Kyoto encyclopedia of genes and genomes (KEGG) pathway analysis of DEPs

**Fig. 3:**
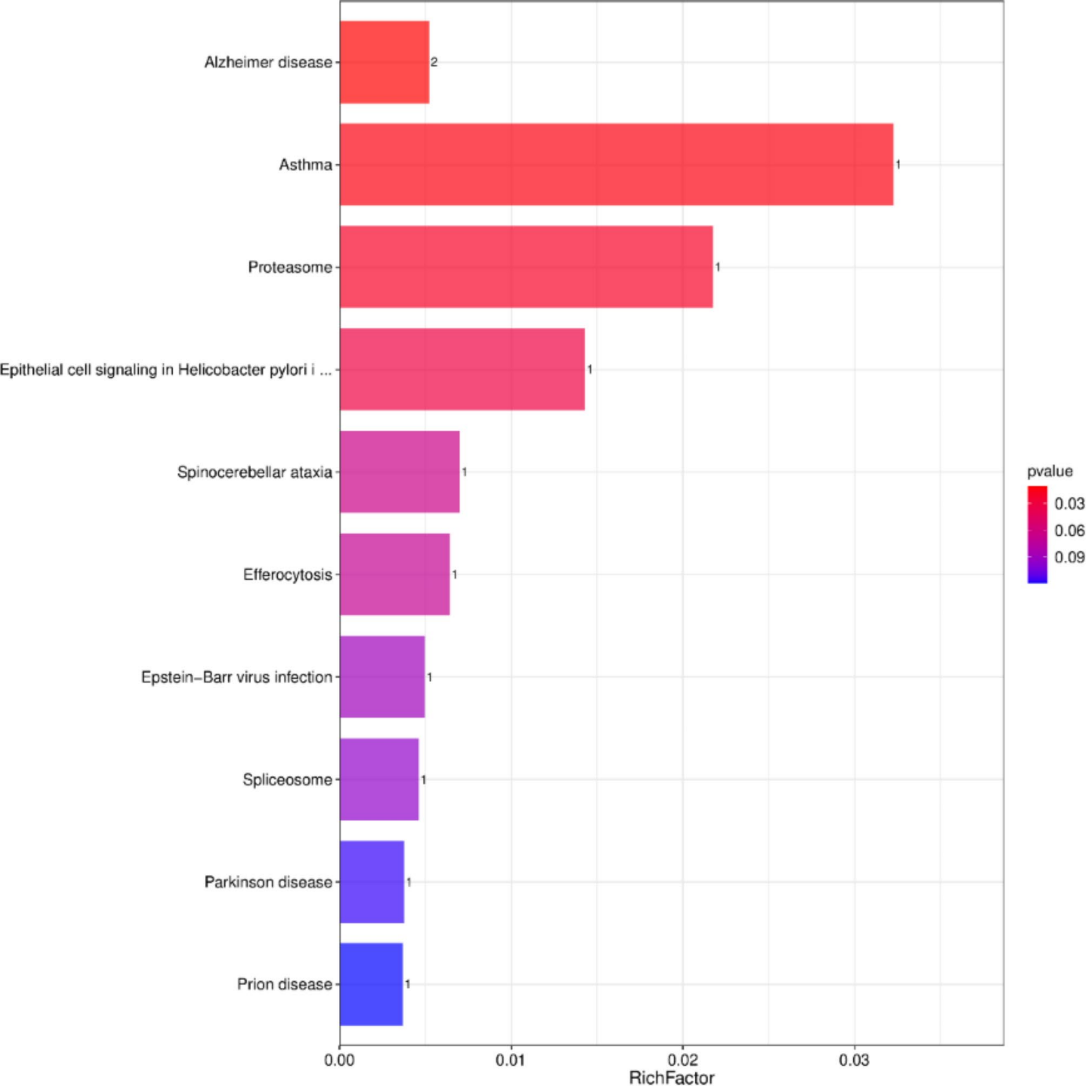
KEGG pathway enrichment analysis of DEPs with the ten highest enrichment scores. The left y-axis shows the pathways. The right y-axis colored with gradient color is P value showing the enrichment score. The greater the enrichment score, the smaller the P value, signifying the significant enrichment of DEPs within the specified pathways.

The KEGG pathway enrichment analysis was conducted to identify the functions of DEPs. The results showed that DEPs in uric stone group compared with the control group were involved in 10 KEGG pathways. As shown in Fig. 3, the top 3 significant pathways were as follows: Alzheimer’s disease, asthma, and the proteasome.

### Metabolomics analysis of urine samples

**Fig. 4:**
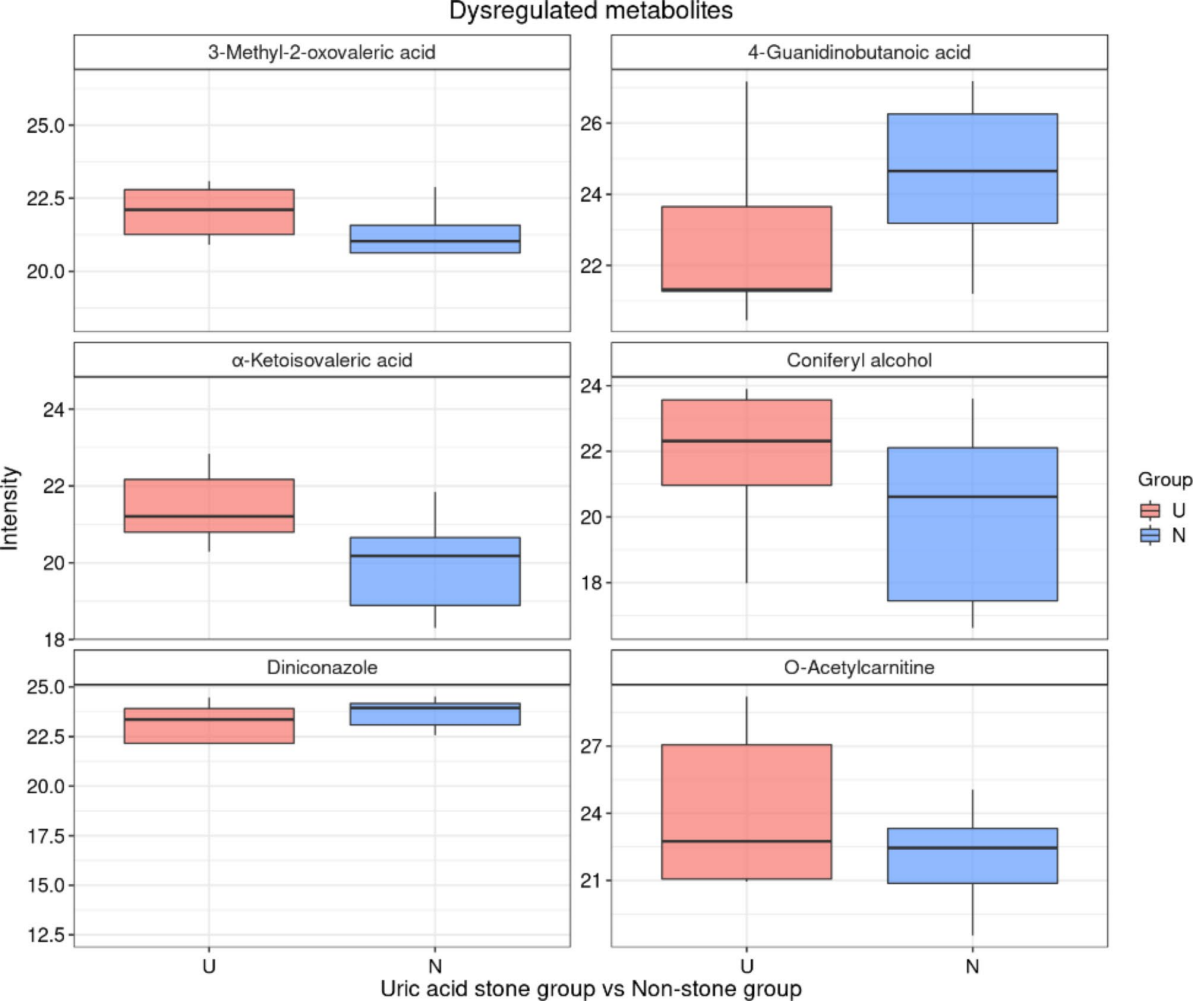
The relative content of dysregulated metabolites in uric acid calculus group and non-stone group.

**Fig. 5:**
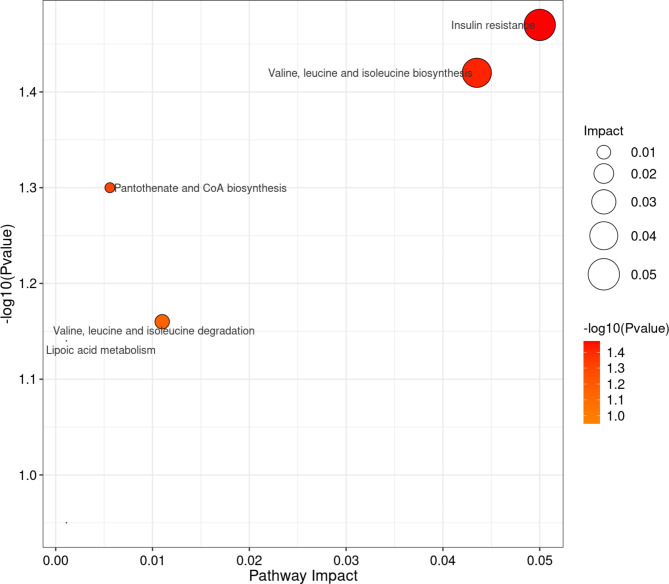
KEGG pathway enrichment analysis of dysregulated metabolites with the four highest enrichment scores. The right y-axis colored with gradient color is -log10 (Pvalue) showing the enrichment score. The greater the enrichment score, the greater the P value, signifying the significant enrichment of dysregulated metabolites within the specified pathways.

A total of 460 metabolites were detected, identified and quantified in the urine of 9 patients with bilateral renal pelvis. Using a dual criterion of *P* < 0.05 and FC > 1.2, we attempted to identify common features among the 9 paired samples. Unfortunately, none of the metabolites met the screening criteria in all nine pairs of samples. However, several dysregulated metabolites aroused our interest (Table 3), all of which exhibited varying degrees of elevation or decrease in uric acid stone urine (Fig. 4). KEGG analysis showed that dysregulated metabolites were mainly associated with insulin resistance and valine, leucine and isoleucine biosynthesis (Fig. 5).

**Table 3:** List of dysregulated metabolites.

Name	Exact mass	Formula	Expressed	*p*-value
O-Acetylcarnitine	204.1236	C9H18NO4	Up	0.000
Coniferyl alcohol	180.0786	C10H12O3	Up	0.048
alpha-Ketoisovaleric acid	116.0473	C5H8O3	Up	0.003
3-Methyl-2-oxovaleric acid	130.063	C6H10O3	Up	0.001
4-Guanidinobutanoic acid	145.0851	C5H11N3O2	Down	0.009
Diniconazole	325.0749	C15H17Cl2N3O	Down	0.002

## Discussion

In previous studies, uric acid stones appeared to be a disease related to systemic metabolism^13,14^. Due to differences in individuals’ diets, ages, underlying conditions, medication usage, and other factors, the proteins and metabolites in the blood and urine vary greatly between individuals. The study of specific biomarkers in patients with uric acid stones requires strict control over the subjects’ dietary and lifestyle habits during research. Additionally, to avoid analysis errors caused by individual differences in metabolic profiles, a large sample size is required. Thus far, no specific serum or urine biomarkers for uric acid stones have been discovered. Our study focuses on patients with unilateral renal uric acid stones, where urinary stone disease has historically occurred only in one kidney of these patients. We hypothesize that there are significant differences in protein and metabolic profiles between the two kidneys of these patients. We use a self-controlled method, which does not require regulating the diet of the study subjects. This effectively avoids errors caused by differences in individual lifestyles, thereby allowing for a more accurate study of the differences in urine protein and metabolic profiles in uric acid stones.

Low urine pH is well established as the primary determinant of uric acid nephrolithiasis^15,16^. Research indicates that the formation of uric acid stones is due to a decrease in renal tubular ammonia production, resulting in an imbalance between renal ammonia production and uric acid excretion, ultimately leading to urine acidification and the precipitation of uric acid crystals^17^. Bobulescu et al.^18^ found excessive organic acids in the urine of patients with idiopathic uric acid stones. These organic acids participate in the tricarboxylic acid cycle, lipid metabolism, and amino acid metabolism. Our results indicate that in the renal pelvis urine of uric acid stone patients, α-ketoisovaleric acid and 3-methyl-2-oxovaleric acid are elevated to varying degrees. These are the metabolic products of valine and isoleucine, respectively^19^. Excessive amounts of α-ketoisovaleric acid and 3-methyl-2-oxovaleric acid in the urine may lead to acidification of the urine, thereby promoting the precipitation of uric acid crystals.

The role of elevated branched-chain amino acids (leucine, isoleucine, and valine) in metabolic diseases is still controversial, but branched-chain amino acids and their derivatives are widely considered to be some of the strongest biomarkers for a range of metabolic diseases, including obesity, insulin resistance, and type 2 diabetes mellitus^20,21^. Research has shown that patients with metabolic syndrome have a much higher probability of developing uric acid stones than the general population, and insulin resistance, hyperlipidemia, obesity, and hyperuricemia are risk factors for uric acid stones^22–25^. Acetylcarnitine is the final metabolite of the beta-oxidation pathway and is crucial for maintaining mitochondrial function and glucose homeostasis in the face of energy surplus^26^. Our study found that the level of acetylcarnitine in the urine of uric acid stone patients was significantly higher than in stone-free urine, suggesting the presence of mitochondria-related metabolic disorders in the bilateral kidneys of unilateral uric acid stone patients. However, whether the elevation of this substance in urine is due to increased renal excretion or underlying renal metabolic abnormalities remains unknown and requires further investigation.

KEGG analysis of urine metabolomics showed significant correlations between dysregulated metabolites in uric acid stone urine and insulin resistance, as well as the synthesis and degradation of branched-chain amino acids. The relationship between insulin resistance and uric acid stones has been well described previously. Insulin resistance may decrease ammonium excretion in the proximal renal tubules, potentially contributing to disturbances in acid-base balance, such as metabolic acidosis. Additionally, impaired ammonium excretion can influence the overall urinary pH and may affect the excretion of other substances, including uric acid, thereby potentially contributing to the formation of kidney stones and other renal complications associated with metabolic disorders^15,27^.

The proteomic identification of urine from patients with uric acid stones has been rarely studied in the past, and we found 8 upregulated differential proteins in uric acid stone urine. Khusid et al. performed KEGG pathway analysis of the urine proteome of pure uric acid stone patients, revealing enrichment of pathways such as ECM-receptor interactions, platelet activation, proteoglycans in cancer, and vitamin digestion and absorption, among others^28^. In contrast, our study yielded markedly different results, with Alzheimer’s disease, asthma, and the proteasome pathway being significantly enriched. Although KEGG analysis indicates that the differential proteins in the urine of uric acid stone patients are related to the aforementioned pathways, the number of differential proteins is small, and the associations are not strong, suggesting they may not reflect the actual pathological process.

Several of these proteins caught our attention. Eosinophil peroxidase (EPX) is an eosinophil-specific cationic protein that is localized in the matrix of the secondary granules and has also been shown to be a useful biomarker of eosinophilic inflammation in a wide variety of clinical samples^29^. Prohibitin 1 (PHB1) associates with the homologous protein PHB2, forming a large oligomeric ring-like structure in the inner mitochondrial membrane, aids in the regulation of several aspects of mitochondrial biogenesis, dynamics, and metabolism^30^. Several recent lines of research also suggest that PHB plays a crucial role in the oxidative balance of tissues. PHB might act as an antioxidant of the mechanisms that counteract oxidative stress and extracellular matrix (ECM) component accumulation in Renal tubular epithelial cells^31,32^. A disintegrin and metalloproteinase domain-containing protein 10 (ADAM10) is highly expressed on the cell surface of epithelial cells in the distal tubule as well as endothelial cells, particularly in diseased endothelium^33,34^. Their main function is the cleavage of a variety of membrane-bound proteins, including many cytokines and growth factors. In a rat model of chronic kidney diseases, increased expression of ADAM10 contributed to epithelial-to-mesenchymal transition of tubular epithelia and increased kidney fibrosis^35^. Moreover, in a mouse model, it could be demonstrated that activation of ADAM10 promoted kidney interstitial fibrosis and eventually renal dysfunction^36^. The role of these proteins in the pathophysiology of the kidney has been partially confirmed, but the role of these proteins in kidney stones has not been demonstrated.

Several other proteins have not been fully studied in renal pathophysiology. Cholinesterase has broad substrate specificity and contributes to the inactivation of the neurotransmitter acetylcholine^37^. Beta/gamma crystallin domain-containing protein 2 was highly expressed in some tumor tissues, including esophageal squamous cell carcinoma and hepatocellular carcinoma^38,39^. Cell division cycle 5-like protein is a DNA-binding protein involved in cell cycle control and acts as a transcriptional activator^40^. 26S proteasome non-ATPase regulatory subunit 11, a component of 26S proteasome, is involved in the ATP-dependent degradation of ubiquitinated proteins^41^. Ficolin-3 may play a role in innate immunity by activating the lectin complement pathway^42^. The pathophysiological effects of the above 5 proteins in kidney need to be further studied.

Therefore, the role of proteins in uric acid stone formation still requires further research. However, using a self-controlled method, we did identify 8 highly expressed proteins in the urine of uric acid stone patients.

Several limitations exist in our study. Firstly, we relied on imaging data such as CT scans and ultrasounds, as well as patient histories, to determine unilateral stone presence, with the possibility of undetected microscopic stones. Additionally, our study was self-controlled and had a small sample size, and it lacked targeted proteomics and metabolomics validation among large sample individuals.

## Conclusion

In conclusion, our research indicates differences in protein and metabolic profiles between the urine of the two kidneys in unilateral uric acid stone patients. In the urine from the uric stone-affected side, eight differential proteins were significantly upregulated, and 6 metabolites were dysregulated. Furthermore, we discovered elevations of α-ketoisovaleric acid and 3-methyl-2-oxovaleric acid in the urine environment of uric acid stone patients, which may be one of the causes of urine acidification.

## Electronic supplementary material

Below is the link to the electronic supplementary material.

Supplementary Material 1

## Data Availability

The datasets used and/or analysed during the current study are available from the corresponding author on reasonable request.
