# Supplementary material for: Self-control study of multi-omics in identification of microenvironment characteristics in urine of uric acid stone
Source: Sci Rep. 2024 Oct 24;14:25165. doi: 10.1038/s41598-024-76054-0 (PMC11502694; doi:10.1038/s41598-024-76054-0)
Supplement: Supplementary file 1 — Supplementary Material 1 [file 41598_2024_76054_MOESM1_ESM.docx]

**Proteomic Materials and Method**s

**1.LC-MS/MS Analysis**

For spectral library generation, samples were fractionated using a high pH reversed-phase fractionator. The mass spectrometer was operated on a quadrupole Orbitrap mass spectrometer (Q Exactive HF-X, Thermo Fisher Scientific, Bremen, Germany) coupled to an EASY nLC 1200 ultra-high pressure system (Thermo Fisher Scientific) via a nano-electrospray ion source. 500 ng of peptides were loaded on a 25 cm column (150 μm inner diameter, packed using ReproSil-Pur C18-AQ 1.9- µm silica beads; Beijing nuomi Biotech Co.,Ltd, Beijing, China). Peptides were separated using a gradient from 8% to 12% B in 7 min ,then12% to 30 % B in 48 min and stepped up to 40% in 10 min followed by a 15 min wash at 95% B at 600 nl per minute where solvent A was 0.1% formic acid in water and solvent B was 80% ACN and 0.1% formic acid in water. The total duration of the run was 80 min. Column temperature was kept at 60 °C using an in-house-developed oven. Briefly, the mass spectrometer was operated in “top-40” data-dependent mode, collecting MS spectra in the Orbitrap mass analyzer (120,000 resolution, 350–1500 m/z range) with an automatic gain control (AGC) target of 3E6 and a maximum ion injection time of 80 ms. The most intense ions from the full scan were isolated with an isolation width of 1.6 m/z. Following higher-energy collisional dissociation (HCD) with a normalized collision energy (NCE) of 27, MS/MS spectra were collected in the Orbitrap (15,000 resolution) with an AGC target of 5E4 and a maximum ion injection time of 45 ms. Precursor dynamic exclusion was enabled with a duration of 16 s.

For Data-independent acquisition（DIA）, the acquisition method consisted of one MS1 scan (350 to 1500 m/z, resolution 60,000, maximum injection time 50 ms, AGC target 3E6) and 42 segments at varying isolation windows from 14 m/z to 312 m/z (resolution 30,000, maximum injection time 54 ms, AGC target 1E6). Stepped normalized collision energy was 25, 27.5 and 30. The default charge state for MS2 was set to 3.

**2.The identification and quantitation of protein**

Mass spectrometry data processing The MS data of the fractionated pools (Data Dependent Acquisition (DDA) MS data, 6 fractions) and the single-shot subject samples (DIA MS data) were used to generate a DDA-library and direct-DIA-library, respectively, which were computationally merged into a hybrid library in the Spectronaut software (Biognosys, version 15.7.220308.50606). The hybrid spectral library was used to search the MS data of the single-shot samples in the Spectronaut software, for final protein identification and quantitation. All searches were performed against the uniprot Homo sapiens SP proteome database (20,407 target sequences downloaded on 2023-03-07). Searches used carbamidomethylation as fixed modification and acetylation of the protein N-terminus, oxidation of methionines as variable modifications. Default settings were used for other parameters. In brief, a trypsin/P proteolytic cleavage rule was used, permitting a maximum of two miscleavages and a peptide length of 7–52 amino acids. Protein intensities were normalized using the “Local Normalization” algorithm in Spectronaut based on a local regression model (Callister et al, 2006). Spectral library generation stipulated a minimum of three fragments per peptide, and maximally, the six best fragments were included. A protein and precursor FDR of 1% were used and protein quantities were reported in samples only if the protein passed the filter (“Q-value sparse” mode data filtering).


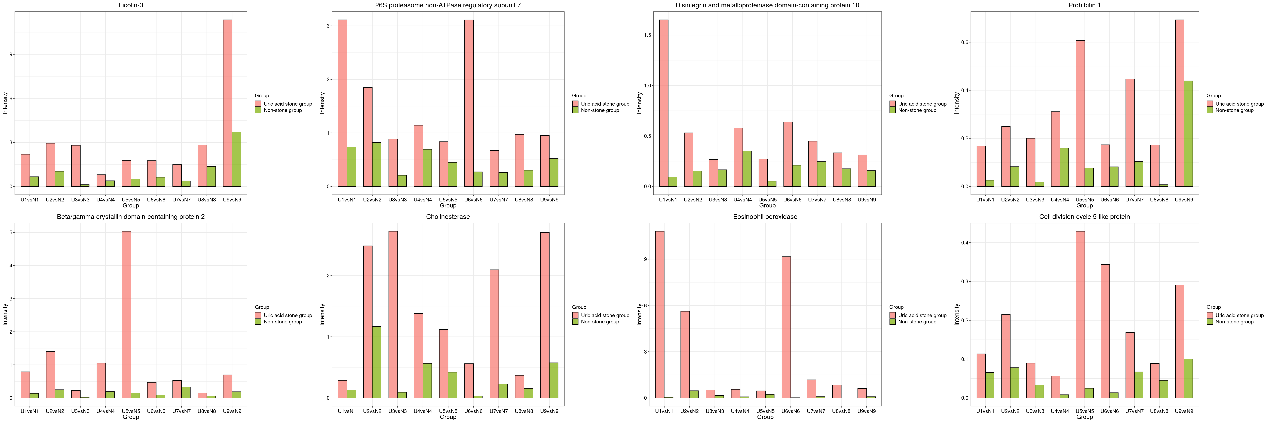


**Figure S1** The relative quantification of the 8 differential proteins in the 9 paired samples.
